# Isolation and morphological and molecular characterization of waterborne free-living amoebae: Evidence of potentially pathogenic *Acanthamoeba* and Vahlkampfiidae in Assiut, Upper Egypt

**DOI:** 10.1371/journal.pone.0267591

**Published:** 2022-07-08

**Authors:** Martina M. Nageeb, Hanan E. M. Eldeek, Rasha A. H. Attia, Atef A. Sakla, Samia S. Alkhalil, Haiam Mohamed Mahmoud Farrag

**Affiliations:** 1 Medical Parasitology Department, Faculty of Medicine, Assiut University, Assiut, Egypt; 2 Department of Clinical Laboratory Sciences, Faculty of Applied Medical Sciences, Shaqra University, Shaqra, Saudi Arabia; University of Limpopo, SOUTH AFRICA

## Abstract

Free-living amoebae (FLA) are gaining attention due to the increasing number of related grave central nervous system (CNS) and sight-threatening eye infections and their role as Trojan horses for many bacteria and viruses. This study was conducted in Assiut City, Egypt to detect the presence of FLA in different water sources using morphological and molecular approaches and determine their potential pathogenicity. A total of 188 water samples (100 tap, 80 tank, and 8 swimming pool samples) were collected, cultivated on non-nutrient agar seeded with *Escherichia coli*, and inspected for FLA. Thermo- and osmo-tolerance assays were performed to determine their pathogenicity. Polymerase chain reaction and sequence analysis were performed to confirm the identification and analyze the genotype. Overall, 52 samples (27.7%) were positive for FLA. Of these, 20.7% were identified as *Acanthamoeba*, 1.6% as Vahlkampfiidae, and 5.3% as mixed *Acanthamoeba* and Vahlkampfiidae. Seven species of *Acanthamoeba* were recognized, of which *A*. *triangularis*, *A*. *polyphaga*, *A*. *lenticulata*, and *A*. *culbertsoni* are thermo- and osmo-tolerant, and *A*. *astronyxis*, *A*. *comandoni*, and *A*. *echinulata* are non-thermo- and non-osmo-tolerant. The phylogeny analysis revealed T4 and T7 genotypes. Among Vahlkampfiids, 61.5% were identified as thermo- and osmo-tolerant *Vahlkampfia*, and 30.8% were identified as non-pathogenic *Naegleria*. One isolate (7.7%) was identified as potentially pathogenic *Allovahlkampfia*, as confirmed by sequencing. This is the first report documenting the occurrence and phylogeny of waterborne FLA (*Acanthamoeba/*Vahlkampfiidae) in Assiut, Egypt. The presence of potentially pathogenic FLA highlights the possible health hazards and the need for preventive measures.

## Introduction

FLA represents a large and diverse group of amoebic protozoa found in a wide range of habitats worldwide. They are often referred to as amphizoic amoebae due to their ability to live freely without a host and their ability to invade the host and survive as parasites [1]. These amoebae feed on bacteria, fungi, algae, and small organic particles in their natural environment. However, some can become tissue feeders and facultative pathogens to humans [2]. All members of the FLA, including *Acanthamoeba* and Vahlkampfiids, are transmitted to humans mainly through different water sources (such as freshwater lakes, swimming pools, therapeutic pools, tap water, seawater, and sewage systems), causing various pathological disorders [3].

Among pathogenic FLA, *Acanthamoeba* cause chronic granulomatous amoebic encephalitis (GAE), which principally affects immune-compromised patients. They also cause amoebic keratitis (AK), which affects contact lens wearers or, infrequently, non-contact lens wearers, resulting in permanent visual impairment or complete blindness [3, 4]. Some *Acanthamoeba* species can cause rhinosinusitis, pulmonary infections [5], cutaneous lesions, and osteomyelitis [6].

Vahlkampfiidae comprises many genera that live in different aquatic habitats worldwide with *Naegleria* and *Vahlkampfia* being most frequently found in the environment. Of the 47 *Naegleria* species, only *N*. *fowleri* is pathogenic to humans, causing acute and fatal primary amoebic meningoencephalitis (PAM). However, other species of *Naegleria* have revealed pathogenicity only in experimental mice [7, 8]. Other genera of the family Vahlkampfiidae, including *Vahlkampfia*, *Allovahlkampfia*, *Vermamoeba*, and *Paravahlkampfia*, have also been isolated from human cases of corneal infections worldwide [9, 10].

In addition to the direct potential pathogenic outcome of FLA infection, their ability to act as a Trojan horse for pathogenic mediators, such as bacteria, fungi, and viruses, has gained much attention [11, 12]. FLA and all the pathogenic agents carried by them are potentially conducted to humans through water sources, causing pathological consequences [3]. Furthermore, FLA is highly resistant to extreme temperature, pH, and exposure to chemicals [13]. This represents challenges in eradicating pathogens from public water supplies, especially in developing countries, like Egypt. The *in vitro* growth of amoeba isolates under relatively high osmotic stress or temperature can be related to their virulence. Thermo-tolerance is correlated to the capability of the amoebae to withstand an average body temperature or even fever episodes in the host [1]. Additionally, osmo-tolerance is associated with the ability to resist high osmotic pressures, a situation that the amoebae could face when they act as parasites of the corneal epithelium to overcome the protective salinity of the tears [14].

Traditionally, *Acanthamoeba* are distinguished beyond the genus level into three groups, incorporating more than 20 species according to cyst size and shape, which were the most appropriate criteria at that time [15]. Recently, a sub-generic identification system has been designated, which involves sequence analysis of the diagnostic fragment three (DF3) region of the ribosomal DNA gene to classify the *Acanthamoeba* strains into sequence types [16]. To date, at least 22 genotypes have been identified [17–19]. Recently, a novel genotype, designated ‘T23’, bearing cyst structure conforming to morphological group III has been identified [20]. The largest and most diverse sequence type is T4, which is most prevalent in both clinical and environmental sources, with multiple infectious strains [21, 22].

There is a lack of data regarding FLA prevalence in aquatic environments in Egypt, with a scarcity of phylogenetic studies [3, 8]. Therefore, this study aimed to determine FLA occurrence in different water sources in Assiut City, Egypt and characterize them using morphological and molecular approaches.

## Materials and methods

### Study area

**Fig 1:**
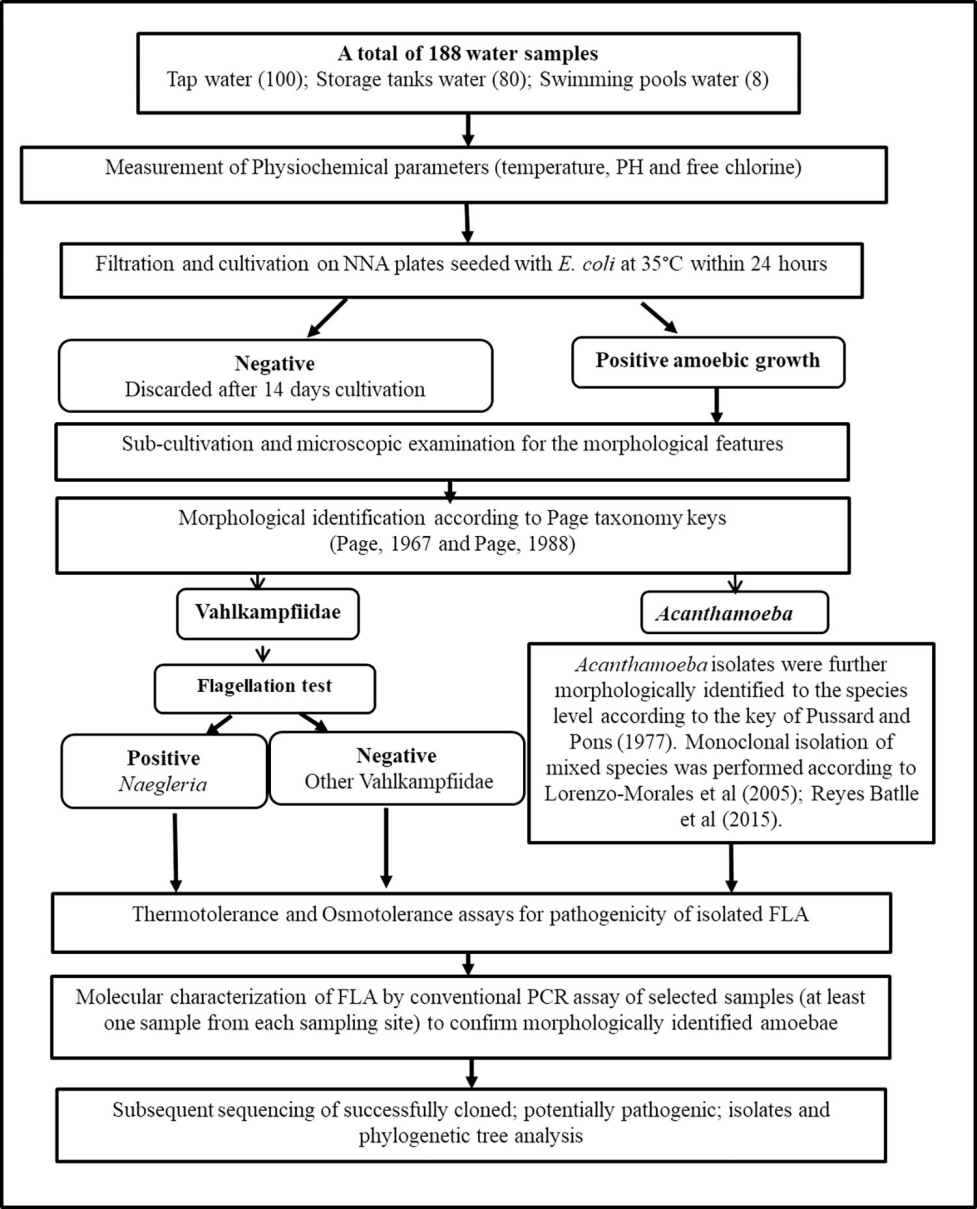
Flow-chart showing the steps of the current study.

This cross-sectional descriptive study was conducted in Assiut City, Assiut Governorate, Upper Egypt, which is located at a latitude of 27° 10’ 51.46" N and longitude of 31° 11’ 1.25" E and has an elevation of 56 m above sea level and an average atmospheric pressure of 1015 hPa at the sea level [23]. According to the Köppen Climate Classification System, the Assiut Governorate is located in a dry, arid region, characterized by virtually no rainfall throughout the year and an average precipitation of 2 mm [24]. Air temperatures from September 2017 to October 2018 have shown minimum to maximum temperatures of 1°C to –45°C, respectively, with a mean average temperature of 25°C, according to the “Weather underground report” "https://www.wunderground.com/weather/eg/asyut."The steps of the current study are summarized in **Fig 1**.

### Sample collection and filtration

A total of 188 water samples were collected from three different water sources in Assiut City, Egypt, including taps (100 representative samples from 25 sampling points), roof storage tanks (80 samples from 20 sampling points), and swimming pools (8 samples from 2 pools) (S1 Table). The samples were collected from September 2017 to August 2018 (all four seasons were included). The same water source in each location was chosen for sample collection in each season. Water samples were collected in 3 L sterile containers labeled with the name of the sampling site and collection date. The samples were then transported to the laboratory of the Department of Parasitology, Assiut University, Egypt, at ambient temperature for further processing on the same day. The water temperature was measured at the sampling site using a water thermometer. In contrast, the pH and free chlorine levels were measured in the laboratory using a pool test kit (Palintest, UK), according to the manufacturer’s specifications. Water samples were then filtered through a cellulose nitrate membrane filter (0.45 μm pore size and 47 mm diameter; Whatman, WCN type, Cat No. 7141–104, Cytiva, USA) fitted to a stainless-steel filter apparatus designed and manufactured by the Faculty of Engineering at Assiut University, as described by Sayed *et al*. (2016) and Hassan *et al*. (2019) [25, 26] (S1 File).

### Culture, subculture, and isolation of FLA

After the filtration process, the filter membrane of each sample was inverted face-to-face on the surface of a non-nutrient agar (NNA) plate (El Gomhouria, Egypt) seeded with heat-killed *Escherichia coli*. The plate was then wrapped and incubated at 35°C to permit the growth and multiplication of FLA existing in the water samples. The culture plates were examined daily by light microscopy to identify amoeba cysts and trophozoites. The plates were considered negative if no amoebic growth was observed after 14 days of incubation and discarded. All positive cultures were submitted to subculturing to obtain the isolates in sufficient quantities. Subcultures were carried out according to the methods of Init *et al*. (2010) [27]. Single clone isolation by dilution in NNA was carried out as previously described [28, 29].

### Morphological identification of FLA

The trophozoites and cysts of different types of FLA were examined under light microscopy (Olympus, Japan) and morphologically analyzed to identify the genus level, according to the keys described by Page (1967) and (1988) [30, 31]. Isolated members belonging to *Acanthamoeba* were further morphologically characterized to the species level according to the key of Pussard and Pons (1977) [15].

A flagellation test was performed for positive culture samples to differentiate the genus *Naegleria* from other FLA, especially the genus *Acanthamoeba* and other members of the family Vahlkampfiidae [27]. The flagellation test was carried out by incubating the amoebae in a test tube containing distilled water for a couple of hours, which was then examined every 10 min for the presence of the flagellated forms [32].

### Thermo- and osmo-tolerance assays

To examine the effect of temperature on the growth of FLA trophozoites, NNA plates seeded with *E*. *coli* were inoculated with 1000 trophozoites counted by a hemocytometer, incubated at 40°C and 42°C, and observed daily for up to 72 h. To detect the effect of osmolarity on the growth of FLA trophozoites, NNA plates containing 0.5 and 1.0 M mannitol seeded with *E*. *coli* were inoculated with 1000 trophozoites on the center of these plates and incubated at 30°C with daily observation for up to 72 h. For both assays, amoeba growth was observed and determined by measuring the increase of the clearance zone in the bacterial lawn at 0, 24, 48, and 72 h [33].

### Molecular characterization of FLA isolates

#### Collection of amoebae

From positive subcultures, the trophozoites and cysts were detached in Page’s amoeba saline, centrifuged at 2,500 rpm for 10 min, and washed at least three times to minimize the presence of *E*. *coli*. Thereafter, clean sediment was used for subsequent DNA extraction and molecular analyses [34, 35].

#### DNA extraction

Genomic DNA was extracted from the suspected isolates using the Qiagen extraction tissue kit (QIAamp^®^ DNA Minikit, Germany), according to the manufacturer’s recommendations. The extracted DNA was quantified using a spectrophotometer (NanoDrop ND-1000, Thermo Fisher Scientific, USA) and stored at −20°C until further use.

#### Polymerase chain reaction (PCR) assay

PCR was conducted to amplify the base pair (bp) segment of the 18S rDNA using the primers JDP1 and JDP2, which amplified the 423–551 bp fragments in the case of *Acanthamoeba* [36]. Genus-specific primers ITS1 and ITS2 were used to amplify the bp segment of the ITS region of Vahlkampfiid amoebae, including *Naegleria*. The species-specific primers Fw1 and Fw2 were used to specifically detect *Naegleria fowleri* [37]. The primer sequences and specificities are shown in **Table 1.**

**Table 1:** The primers ’sequences used in PCR reaction in the present study.

Gene	Primer sequence	Specificity	PCR (base pair)	Reference
ASA.S1 (DF3)	**JDP1** 5′ -GGCCCAGATCGTTTACCGTGAA-3′	***Acanthamoeba***	**423 to 551 bp**	[36]
	**JDP2** 5′-TCTCACAAGCTGCTAGGGGAGTCA-3′			
ITS	**ITS1**: F 5′-GAACCTGCGTAGGGATCATTT-3′	***Naegleria***	**400 bp**	[37]
		***Allovahlkampfia***	**500 bp**	
	**ITS2**:R 5′-TTTCTTTTCCTCCCCTTATTA-3′	***Vahlkampfia***	**600 bp**	
		***Hartmannella***	**800 bp**	
ITS	**Fw1:** (5’GTGAAAACCTTTTTTCCATTTACA-3’)	***Naegleria fowleri***	**310 bp**	[37]
	**FW2** (5’AAATAAAAGATTGACCATTTGAAA-3’)			

In the case of *Acanthamoeba* sp., the PCR reactions were carried out in a total volume of 50 μL, which consisted of 25 μL of MyTaq red mix (Bioline, UK), 2 μL of each primer, 4 μL of template DNA, and 17 μL of H_2_O (RNase/DNase-free). PCR amplification was performed in the Veriti Thermal Cycler (Applied Biosystems, USA) programmed with an initial denaturation step at 95°C for 7 min, followed by 45 cycles of denaturation at 95°C for 1 min, annealing at 60°C for 1 min, and extension at 72°C for 2 min; and a final extension step at 72°C for 10 min [36]. However, for Vahlkampfiids and *Naegleria sp*., the PCR reactions were carried out in a total volume of 20 μL, which consisted of 10 μL of MyTaq red mix (Bioline, UK), 1 μL of each primer, 1 μL of template DNA, and 7 μL of H_2_O (RNase/DNase-free). The PCR program was an initial denaturation step at 94°C for 5 min followed by 35 cycles of denaturation at 94°C for 30 s, annealing at 55°C for 30 s, an extension step at 72°C for 45 s, and a final extension step at 72°C for 5 min [37]. Each amplification run contained a negative control (DNase-free water).

The amplification products were analyzed by gel electrophoresis using 1.5% agarose in Tris-acetate EDTA buffer, stained with ethidium bromide (50 μL/L), and visualized on a UV trans-illuminator (Multi Doc-It, UVP, UK). A 100 bp DNA ladder was used to determine the size of the PCR products.

#### Nucleotide sequencing and phylogenetic analysis

A Zymoclean Gel DNA Recovery Kit (Zymo Research, USA) was used to purify PCR products from gel for sequencing. Purified PCR products were sequenced using the same amplification primers used in PCR reactions, and DNA sequencing was carried out by the Macrogen Company (Seoul, South Korea). To determine the genotypes and species of the investigated isolates, the sequences and metadata of 47 *Acanthamoeba* and 10 *Allovahlkampfia* isolates were retrieved from the nucleotide database of the NCBI (https://www.ncbi.nlm.nih.gov/nucleotide/). These sequences were saved in a FASTA file format and used as inputs in MegAlign (a lasergene seven module) (https://www.dnastar.com/software/lasergene/) (S2 Table). The Clustal W method was used for multiple sequence alignment [38], and the phylogenetic tree was constructed after performing a 1000 bootstrap trial. In the *Acanthamoeba* phylogenetic tree, *Allovahlkampfia* was used as an out-group.

### Ethical considerations

Ethical clearance was obtained from the Ethical Committee of the Faculty of Medicine at Assiut University, approving the research protocol and procedures (No: 17100773).

### Statistical analysis

Data were analyzed using SPSS version 20 (IBM, Armonk, New York, USA). Categorical variables were described by number and proportion, where continuous variables were summarized using range, mean, and standard deviation. A comparison of results was made by the Chi-square test, and the *P*-value was considered significant if *P* < 0.05.

## Results

**Fig 2:**
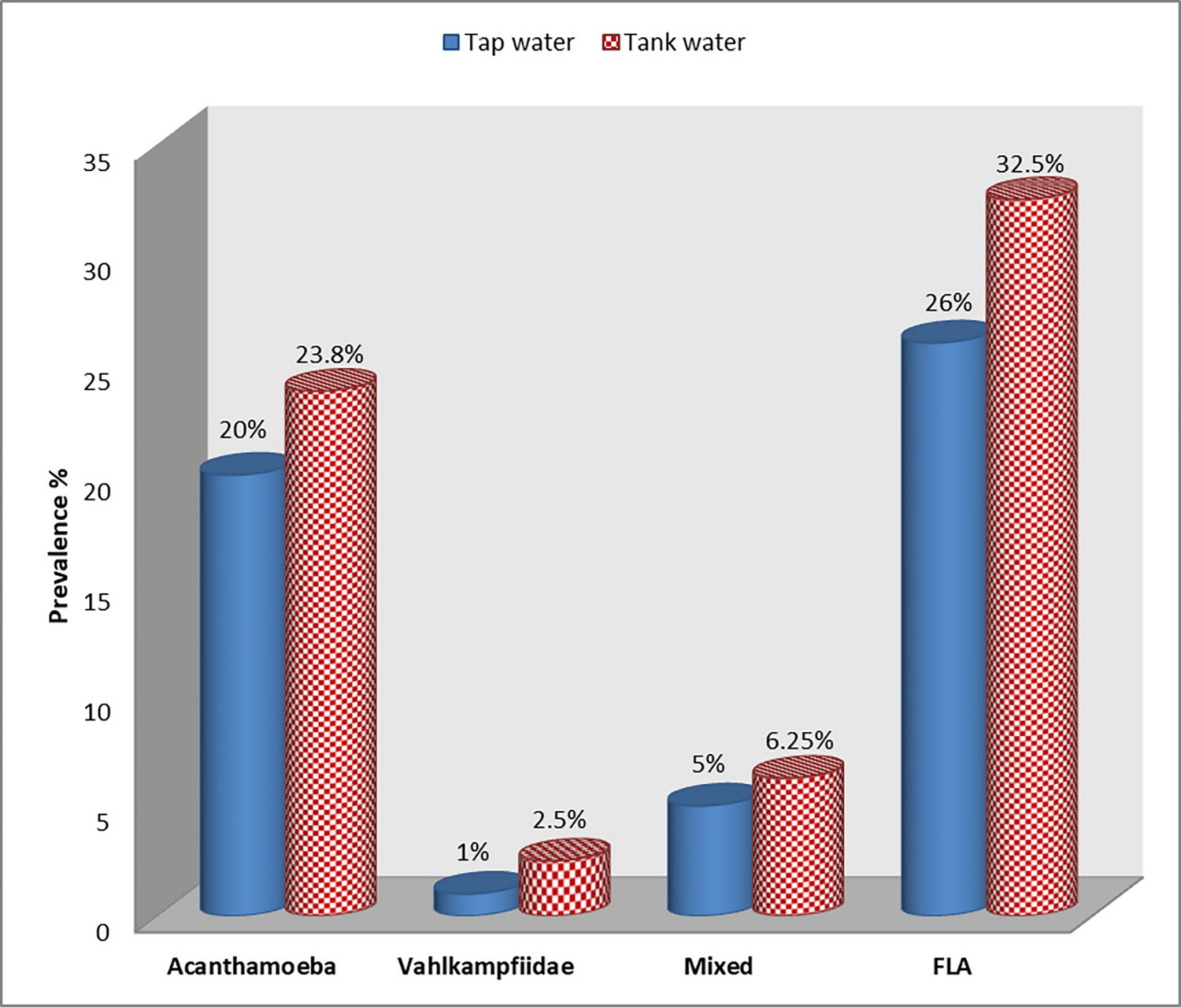
Prevalence of FLA, *Acanthamoeba*, Vahlkampfiidae (*Naegleria*-like), and mixed genera in tap and tank water samples.

Of the 188 water samples collected from different localities in Assiut City in 1 year, 52 (27.7%) samples tested positive for FLA. The prevalence of *Acanthamoeba* was 20.7% (39 positive samples) followed by a 1.6% (three positive samples) prevalence rate for Vahlkampfiidae, while the prevalence rate for mixed *Acanthamoeba* and Vahlkampfiidae was 5.3% (10 positive samples). FLA was detected in 26% and 32.5% of tap and tank water samples, respectively, with no statistically significant difference between the two sources (*P =* 0.127). In contrast, no FLA was detected in any sample collected from the swimming pools. Regarding FLA in different water sources, the highest prevalence rates detected for *Acanthamoeba* were 25% (20% as the single contamination of water samples in addition to 5% as mixed contamination with Vahlkampfiidae) in tap water and 30% (23.8% as single contamination in addition to 6.25% as mixed) in tank water, followed by a prevalence rate of 6% and 8.75% for Vahlkampfiidae in tap and tank water samples, respectively **(Fig 2).**

**Fig 3:**
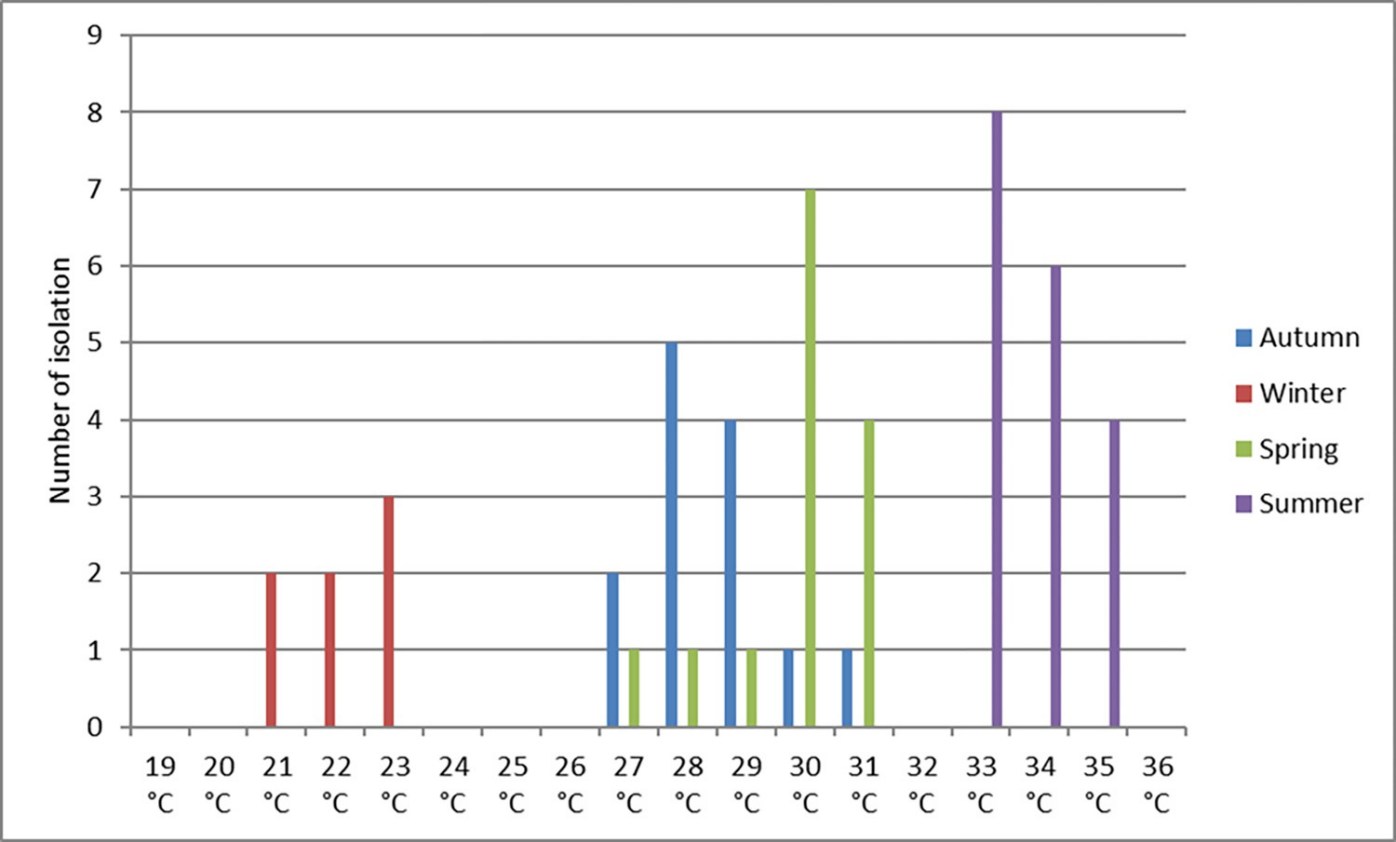
Correlation between the frequency of FLA and water temperature at the sampling time.

The present study recorded the highest prevalence of FLA, *Acanthamoeba* and Vahlkampfiidae, in the summer. In contrast, the lowest prevalence was recorded in winter with no statistically significant variation (*P* > 0.5) (S3 Table). No significant difference was observed in the prevalence of FLA when correlated to the difference in all the water quality parameters in different seasons, including temperature (ranging between 19°C and 36°C), pH (7.4–7.9), and residual chlorine (0.44–0.54). Even though no significant correlation was observed between the FLA detection and water temperature at the time of sampling, there was a trend of increasing amoeba detection with increasing water temperature **(Fig 3)**.

### Morphological characterization of isolated FLA

#### Acanthamoeba

**Fig 4:**
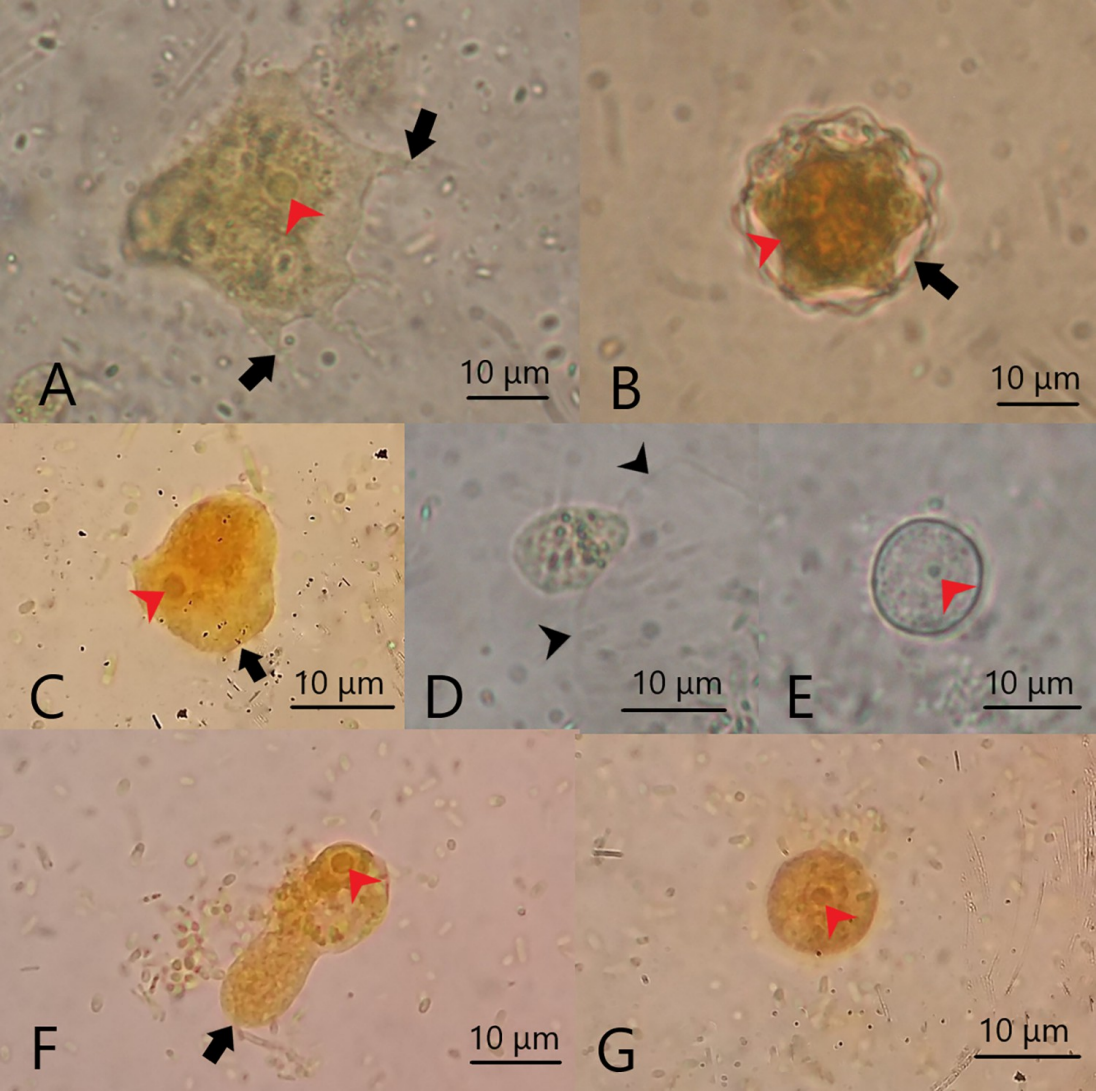
A. *Acanthamoeba* trophozoite showing characteristic acanthopodia (black arrows) with a single nucleus (red arrowhead). B. *Acanthamoeba* cyst showing an outer wall “ectocyst” (black arrow), inner wall "endocyst" (red arrowhead). C. *Naegleria* trophozoite showing blunt hemispherical bulge (lobopodium) at the anterior end (black arrow) and a single nucleus (red arrowhead). D. *Naegleria* flagellate stage, temporary pear-shaped with a pair of flagella (black arrows). E. *Naegleria* cyst with a single nucleus (red arrowhead). F. Vahlkampfiidae trophozoite showing blunt lobopodium (black arrow) and a single nucleus (red arrowhead). G. Vahlkampfiidae cyst with a single nucleus (red arrowhead). (D, E) unstained (A, B, C, F, G) stained with Lugol’s iodine stain. All images are at x1000.

*Acanthamoeba* trophozoites were easily identified by the unique and characteristic presence of fine, tapering, thorn-like acanthopodia that arise from the body’s surface. They were irregular in shape and ranged in size from 25 to 50 μm, depending on the species. In contrast, the cyst stages were double-walled and ranged in size from 11 to 25 μm. The ectocyst was mostly wrinkled with folds while the endocyst varied in shape (stellate, polygonal, oval, or spherical), depending on the species **(Fig 4A and 4B).**

**Fig 5:**
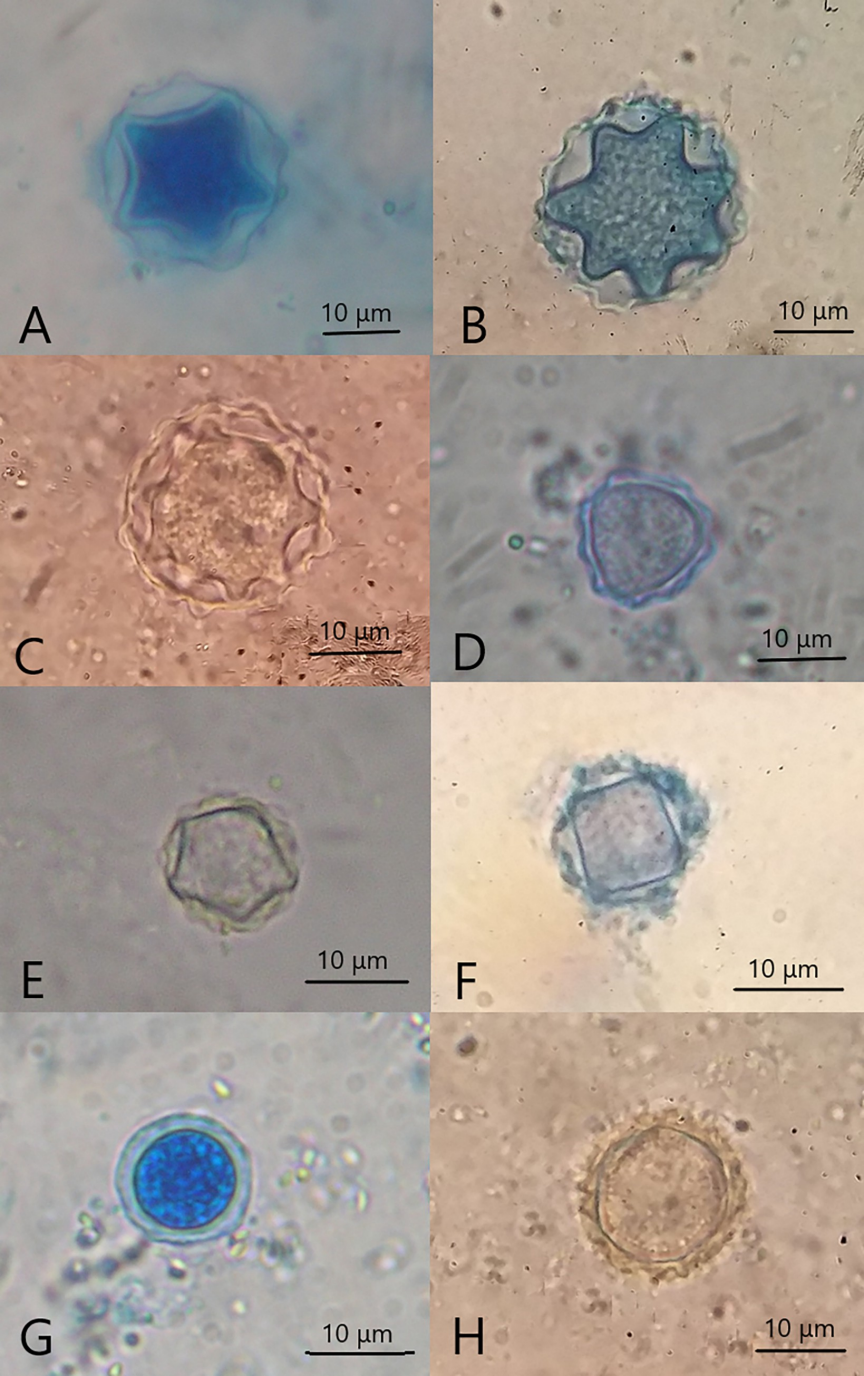
*Acanthamoeba* cysts X1000 A. *A*. *astronyxis*, showing an average cyst diameter of 19–22 μm. Ectocyst was smoothly circular, while the endocyst was stellate with mainly five rays. B. *A*. *comandoni*, showing an average cyst diameter of 20–25 μm, stellate endocyst with mainly 6–9 rays, thin and little wrinkled ectocyst encircling the endocyst. C. *A*. *echinulata*, showing an average diameter of 25 μm, stellate endocyst with a higher number of rays (up to 12–14) and thin, little wrinkled ectocyst. D. *A*. *triangularis*, showing an average diameter of 13 μm. The endocyst was triangular with broad rays. The ectocyst was thick, wrinkled, and corrugated but not spherical. (E, F) *A*. *polyphaga*, showing an average cyst diameter of 14 μm. The endocyst was irregularly polyhedral, usually quadrangular or pentagonal. The ectocyst was folded and loosely applied to the endocyst. G. *A*. *lenticulata*, showing a cyst diameter of 11–13 μm. The endocyst was nearly rounded. The ectocyst closely followed the endocyst contour and pleated forming saw-teeth appearance all-around the endocyst. H. *A*. *culbertsoni*, showing a cyst diameter of 15–18 μm. The ectocyst was more wrinkled and thinner than the endocyst. The endocyst was nearly rounded or with only slight angles. [Cysts of A, B, C belonged to the group (I); D, E, F belonged to the group (II); G and H belonged to the group (III)]. (C, H) unstained (A, B, D, F, G) stained with lactophenol cotton blue stain.

Seven *Acanthamoeba* species were morphologically identified according to the cyst characteristics, namely *A*. *astronyxis*, *A*. *comandoni*, and *A*. *echinulata*, belonging to group (I); *A*. *triangularis* and *A*. *polyphaga*, belonging to group (II); and *A*. *lenticulata* and *A*. *culbertsoni*, belonging to group (III) **(Fig 5A–5H).**

Notably, almost all of the morphologically identified species of *Acanthamoeba* were mixed in the same culture plate of the same sample. All samples positive for *Acanthamoeba* were mixed with their different species. Subculturing helped isolation; however, despite our hard trials, only five samples were successfully mono-isolated. The other subcultured samples showed a predominance of a specific species.

#### Vahlkampfiidae (*Naegleria-*like)

Distinguishing Vahlkampfiid trophozoites and cysts from each other was challenging. The size of each trophozoite ranged from 15 to 20 μm. A distinct anterior hyaloplasmic rim, clearly differentiated from granuloplasm, was observed during active movement. Multiple smooth hyaloplasmic outbursts were detected when changing the direction of movement. The cysts were spherical, measuring 8–15 μm. The cyst walls were thin, with no distinct division into endocyst and exocyst **(Fig 4F and 4G)**.

The *Naegleria* species had three stages; trophozoites, cysts with a double cyst wall (a thick endocyst and a closely opposed thin ectocyst), and characteristic temporary pear-shaped flagellates that ranged in length from 10 to 16 μm with two flagella at the pointed end **(Fig 4C–4E)**.

Of the 13 Vahlkampfiid isolates subjected to the flagellation test, only four (30.8%) produced the flagellate form and were identified as belonging to the genus *Naegleria*. The other nine samples (69.2%), in which the flagellated form could not be observed, were identified as other Vahlkampfiids. In the present study, 10 samples, including the four samples of *Naegleria*, were found mixed with *Acanthamoeba*.

### Thermo- and osmo-tolerance assays of FLA isolates

Regarding the thermo-tolerance assay, *A*. *polyphaga*, *A*. *triangularis*, *A*. *lenticulata*, and *A*. *culbertsoni* isolates were thermophilic at both 40°C and 42°C, whereas *A*. *astronyxis*, *A*. *comandoni*, and *A*. *echinulata* were neither thermotolerant at 40°C nor at 42°C. Concerning the osmo-tolerance assay, all *Acanthamoeba* isolates were osmotolerant at 0.5 M mannitol, while only four species were found to be osmotolerant at 1.0 M mannitol, including *A*. *polyphaga*, *A*. *triangularis*, *A*. *lenticulata*, and *A*. *culbertsoni*.

None of the *Naegleria* isolates were found to be thermotolerant at either 40°C or 42°C, while all other Vahlkampfiidae isolates were able to grow at both 40°C and 42°C. Regarding the osmo-tolerance assay, all *Naegleria* isolates were shown to be osmotolerant at 0.5 M mannitol, while none could grow at 1.0 M. In contrast, the other Vahlkampfiidae isolates were osmotolerant at both 0.5 and 1.0 M mannitol.

### Molecular characterization of FLA isolates

**Fig 6:**
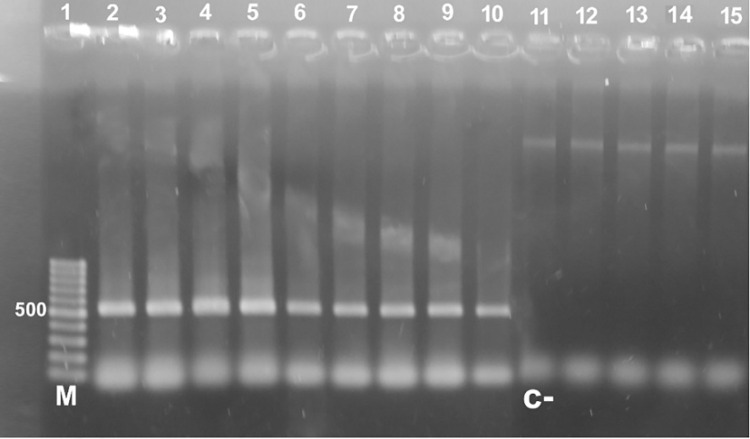
Agarose gel 1.5% stained with ethidium bromide showing the PCR products of 9 positive samples amplifying ASA.S1 region of *Acanthamoeba* using JDP1-2 primers showing a single band of 550 bp. in lanes (2–10), lanes (12–15) are negative samples, lane 11 = negative control, and lane 1 (M) marker = 100 bp DNA ladder.

All *Acanthamoeba* isolates were confirmed to belong to the genus *Acanthamoeba* when examined by PCR by producing amplicons with the expected size at 550 bp through amplification of the ASA.S1 (DF3) region of 18S (r) RNA **(Fig 6)**.

**Fig 7:**
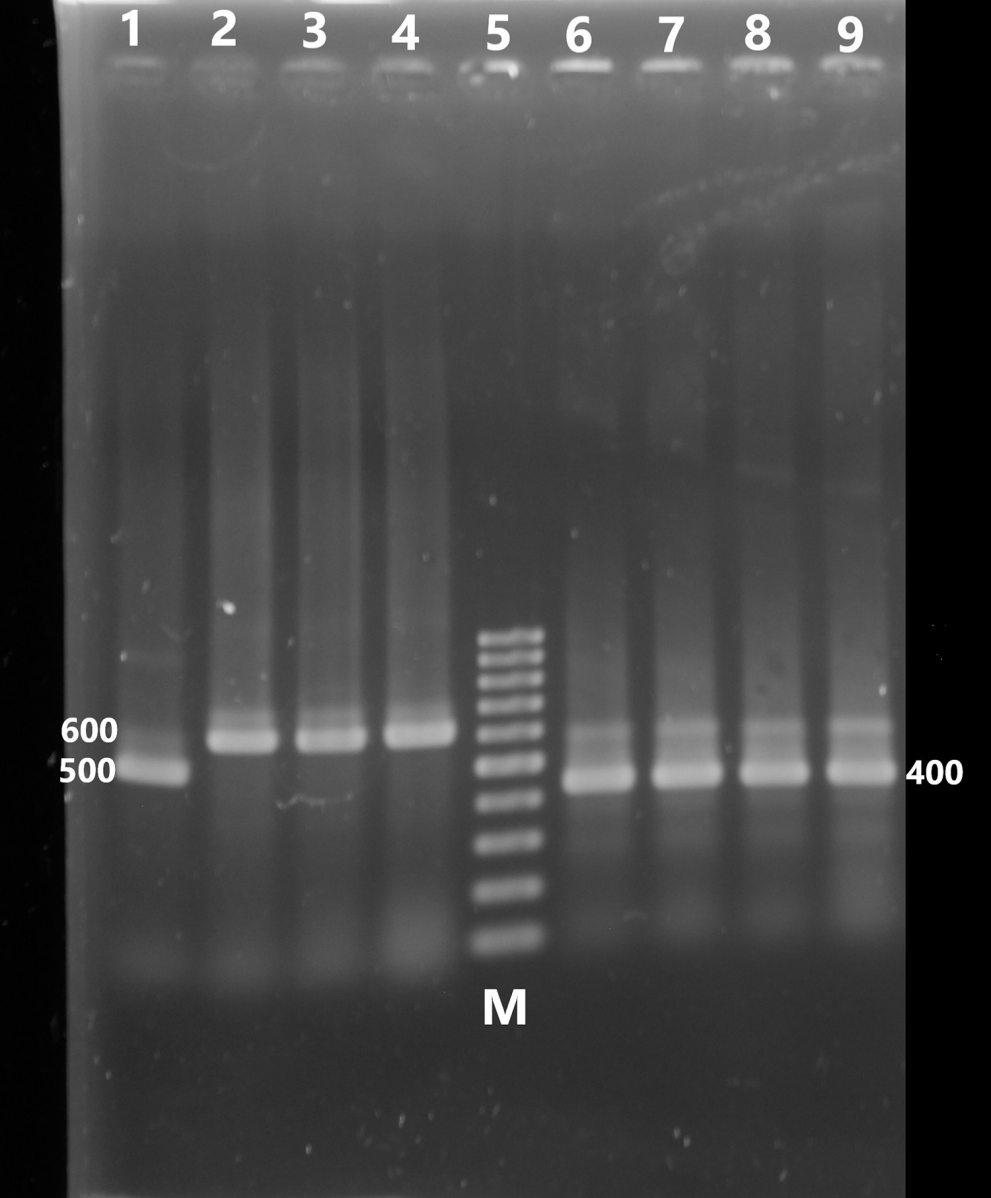
Agarose gel 1.5% stained with ethidium bromide showing the PCR products of 8 positive samples amplifying ITS region of Vahlkampfiidae using ITS1-2 primers showing a single band of 600 bp. (*Vahlkampfia*) in lanes (2,3,4), a single band of 500 bp. (*Allovahlkampfia*) in lane (1) and a single band of 400 bp. (*Naegleria*) lanes (6, 7, 8, 9). Lane 5; (M) marker = 100 bp DNA ladder.

When the 13 Vahlkampfiidae isolates were subjected to PCR amplification of the ITS region using primer sets ITS1 and ITS, eight isolates were confirmed to be related to the genus *Vahlkampfia* by producing amplicons with the expected size of 600 bp. One isolate produced a band of 500 bp and was identified as *Allovahlkampfia*. The other four samples produced bands at 400 bp and were confirmed to be related to the genus *Naegleria* **(Fig 7).** They failed to produce any bands at the expected size of 310 bp using *N*. *fowleri* species-specific primers (S1 Fig).

### Sequencing and phylogenetic analysis

Of the 25 isolates sequenced (at least one isolate from each sampling site, including 16 *Acanthamoeba* and nine Vahlkampfiidae), only six (mono-isolates) were successfully genotyped and analyzed. Phylogenetic analyses showed two *Acanthamoeba* genotypes; T4 and T7.

*A*. *polyphaga* (accession numbers MN091838 and MN091860) and *A*. *culbertsoni* (accession number MN091845) were clustered with isolates of genotype four since they have > 90% similarity with the sequences of isolates belonging to T4. One of the *A*. *polyphaga*, although clustered with T4, showed some degree of similarity (> 90%) with the isolates of T3. *A*. *astronyxis* (accession number MN091379) was clustered very closely to isolates of genotype 7 (similarity = 78%) with high bootstrap values of 98.7%. *A*. *culbertsoni* (accession number MN091854) showed some similarity to T3 and T11, although it showed the highest similarity to genotype four isolates (S2 Fig and S2 File).

**Fig 8:**
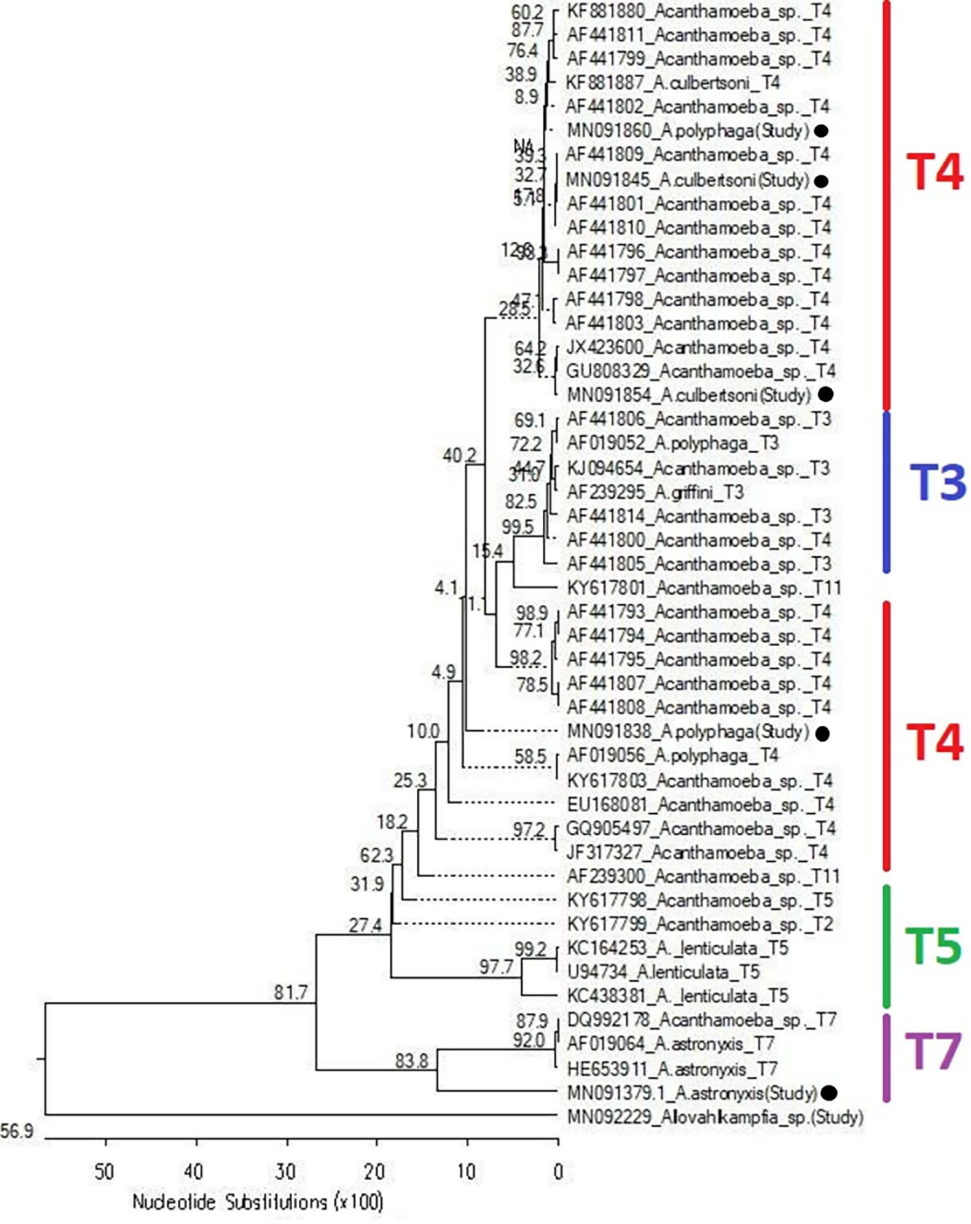
Phylogenetic tree showing the evolutionary relationship among the investigated isolates. Sequences with black circles are the ones analyzed in the current study. The scale below shows the number of substitutions in 100 nucleotides and corresponds to the branch length. Dashed branches indicate negative branches. The values at bifurcations show the node support value in the 1000 bootstrap trials. NA indicates the absence of bootstrapping for the respective node.

**Fig 9:**
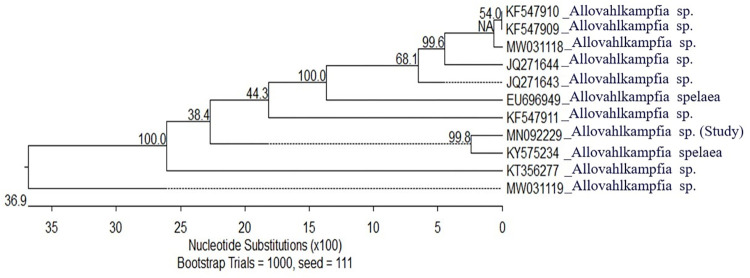
Phylogenetic tree showing the evolutionary relationship among *Allovahlkampfia* spp. The scale below shows the number of substitutions in 100 nucleotides and corresponds to the branch length. Dashed branches indicate negative branches. The values at bifurcations show the node support value in the 1000 bootstrap trials. NA indicates the absence of bootstrapping for the respective node.

Only one isolate of Vahlkampfiidae was successfully genotyped and confirmed to be *Allovahlkampfia* species (accession number; MN092229**)**. The *Allovahlkampfia* sp. was separated from all other analyzed sequences, indicating a vast difference between this species and other *Acanthamoeba* (distances to other species were in the range of 39.8–43.9). The analyzed *Allovahlkampfia* species was found to be closely related to *A*. *spelaea* (accession number KY575234), sharing the same clade with a highly trusted node (99.8%) and a similarity of 95.4%. Meanwhile, our *Allovahlkampfia* spp. showed a similarity of 80.3% to another *A*. *spelaea*. This suggests that our species is potentially *A*. *spelaea* (S3 Fig). However, analyzing full-length genes and including additional isolates would solidify these conclusions. The phylogenetic trees (constructed with sequences of reference species from NCBI-BLAST) and accession numbers are presented in **Figs 8, 9 and Table 2**.

**Table 2:** Genotyping results and water source of FLA mono-isolates in the present study (n = 6).

No.	Isolate ID	Morphological identification	Genotype	Water source	Accession number in GenBank
**1-**	5860057 AST.Eg.2018-1	***A*. *astronyxis***	**T7**	Tap water	**MN091379**
**2-**	5860153 AST.Eg.2018-3	***A*. *polyphaga***	**T4**	Tap water	**MN091838**
**3-**	5860187 AST.Eg.2018-4	***A*. *culbertsoni***	**T4**	Tap water	**MN091845**
**4-**	5860208 AST.Eg.2018-5	***A*. *culbertsoni***	**T4**	Tank	**MN091854**
**5-**	5860250 AST.Eg.2018-8	***A*. *polyphaga***	**T4**	Tank	**MN091860**
**6-**	5860278 AST.Eg.2018-9	***Allovahlkampfia* sp.**		Tank	**MN092229**

## Discussion

With the continually growing number of FLA infections and the significant role of FLA within ecosystems, studies aiming to identify the potential sources of FLA are progressively increasing worldwide, especially those involving environmental waters [39]. In the present study, of 188 collected water samples, 52 were positive for FLA with an overall prevalence of 27.7%. Previous studies conducted in Egypt have recorded higher prevalence rates of 81.4% in Assiut and 86.3% in Fayoum Governorates [40, 41]. The difference in the detection rate of FLA among different countries or even within the same country could be due to the different surroundings and distribution system environment. It may also depend on the source, number, and volume of the collected samples [42]. Moreover, there is no agreement on the method that should be followed to concentrate FLA from water samples, and it varies from a cotton swab to centrifugation or filtration of water samples [43]. Several studies have revealed that the prevalence of FLA can also be affected by the accumulation of biofilms in the water distribution system [44, 45].

A higher prevalence rate of FLA was recorded in tank water samples (32.5%) compared to tap water samples (26%) (*P* > 0.05). This might be explained by the fact that the quality of tank water depends on storage time, physical condition and cleansing of tanks, and different sources that could contaminate tanks, especially those with unfittingly designed sealed tank covers. It was previously described that water storage tanks support FLA colonization, which is consistent with our results [46]. Higher prevalence rates of FLA in tap water samples were recorded in other studies in Egypt [41, 47, 48]. This can be attributed to the time of the study (during summer seasons) and total dissolved solids in water that offer nutrition and a habitat for FLA. Moreover, the difference in FLA prevalence in tap water in different countries worldwide could be attributed to variations in the water source, water treatment method, geographic location, and water temperature [32].

In this study, the genus *Acanthamoeba* was the most prevalent FLA and was present in 25% and 30% of the tap water and tank water samples, respectively. The predominance of *Acanthamoeba* species in our study might be due to their ability to live in all water types and tolerate various weather changes as *Acanthamoeba* cysts were proven to withstand desiccation for more than 20 years [49]. In Egypt, other studies recorded *Acanthamoeba* spp. at different frequency rates in examined water samples [3, 41, 47, 50, 51]. The discrepancies between the results could be attributed to the variation in the source of water samples and the sampling time.

The lower prevalence rate of Vahlkampfiidae (6.9%) in the total examined water samples compared to *Acanthamoeba* may be explained by the weaker ability to survive in adverse conditions beyond 6 months; and the higher sensitivity of its cysts to chlorination [8]. The highest occurrence of FLA, *Acanthamoeba*, and Vahlkampfiidae was recorded in summer with no statistically significant difference between seasons. In agreement with our results, Morsy *et al*. (2016) and Al-Herrawy *et al*. (2017) documented the highest levels of FLA in the summer season [47, 48]. Al-Herrawy *et al*. (2017) also documented the highest levels of *Acanthamoeba* species in tap water samples in the same season [47]. Moreover, our results agree with those of Sifuentes (2012) and Sente *et al*. (2016), who reported the highest prevalence of *Naegleria* in the warmer months of the year [52, 53]. The lack of statistical significance for seasonal variations in FLA detection in the present study is primarily due to the generation of highly resistant cysts that prevail during the different seasons of the year [54].

In the present study, seven species of *Acanthamoeba* were morphologically recognized, namely, *A*. *astronyxis*, *A*. *comandoni*, *A*. *echinulata*, *A*. *triangularis*, *A*. *polyphaga*, *A*. *lenticulata*, and *A*. *culbertsoni*. Various *Acanthamoeba* species have been reported in different studies in Egypt [47, 50, 55] and worldwide [56–58].

The identification of FLA based on morphological observation remains difficult and time-consuming. Therefore, conventional PCR has been developed to be a reliable method for FLA identification [39, 59]. Therefore, the morphologically identified FLA were subjected to molecular approval by the conventional PCR technique using the highly specific primers for *Acanthamoeba* and Vahlkampfiidae, according to the methods of Schroeder *et al*. (2001) and Pélandakis and Pernin (2002), respectively [36, 37]. All morphologically identified *Acanthamoeba* were confirmed to be related to the genus *Acanthamoeba*. For Vahlkampfiidae, *N*. *fowleri* was not detected in our study, a finding that agrees with those of Al-Herrawy *et al*. (2017) and El-Badry *et al*. (2018) [8, 60]. The low cultivation temperature (35°C) may be incriminated in the absence of *N*. *fowleri*. The actual absence of *N*. *fowleri* can also be suspected as, to date, no clinical cases of PAM have been reported in Egypt [8]. Also, in agreement with our results, *Vahlkampfia* was previously isolated from Nile water, Egypt [8].

Thermo- and osmo-tolerance of the isolates were evaluated. Regarding *Acanthamoeba* isolates, *A*. *polyphaga*, *A*. *triangularis*, *A*. *lenticulata*, and *A*. *culbertsoni* were suggested to be potential pathogens as they were thermotolerant at 40°C and 42°C as well as osmotolerant at both 0.5 and 1 M mannitol. On the contrary, *A*. *astronyxis*, *A*. *comandoni*, and *A*. *echinulata* were found to be non-thermotolerant at both 40°C and 42°C and only grew at 0.5 M mannitol. Consequently, they were considered weak potential pathogens [61].

None of the *Naegleria* isolates grew at 40°C and 42°C; thus, we assumed that all *Naegleria* detected were from the non-pathogenic species. Moreover, all other Vahlkampfiidae isolates were found to be osmotolerant at both 0.5 and 1.0 M mannitol and grew at both 40°C and 42°C, and were suggested to be potentially pathogenic. These two characteristics give the amoeba adaptative advantages when parasitizing a host. Growth at high mannitol concentrations has been associated with the ability to resist high osmotic pressures, a situation that the amoebae could face when they act as parasites of the corneal epithelium to overcome the protective salinity of the tears [14]. It is worth mentioning that although some thermotolerant isolates will possibly not be pathogenic, they have great epidemiological importance due to the capability of these amoebae to perform as host vehicles of pathogenic agents, protecting them from adverse conditions [62]. Therefore, after confirmation of the positive FLA isolates (*Acanthamoeba* and Vahlkampfiidae) by PCR and after performing the thermo- and osmo-tolerance assays, we used sequence analysis to confirm the identification and prove the pathogenicity.

In this study, almost all positive samples for FLA were found to be mixed with different species of morphologically identified *Acanthamoeba* and Vahlkampfiidae. These samples were subjected to subcultures several times in a trial for isolation. Mono-isolation was successful for only six isolates. However, the others showed a predominance of certain species (mostly *A*. *polyphaga*, *A*. *culbertsoni*, and *A*. *astronyxis*). Sequencing was performed but unfortunately was successful for only the mono-isolated samples. This situation of mixed species in every single sample and the difficulty of isolation might be attributed to the method used for the collection and filtration of water samples, which permitted the assembly of numerous different species of FLA, as well as the persistent nature of amoeba cysts of many species in every single sample.

Till date, to the best of our knowledge, no studies in the Assiut Governorate have focused on the characterization and genotyping of waterborne *Acanthamoeba* species. In the current study, *Acanthamoeba* isolates belonged to T4 and T7 genotypes, as confirmed by sequencing results. Both genotypes have been reported as causative agents of *Acanthamoeba*-related pathologies. In Egypt, other *Acanthamoeba* types were detected in different studies: T2 and T4 were detected in the Beni–Suef Governorate [3]; T1-4 and T7 were detected in freshwater samples in the Nile Delta [55]; and T3-5, T11, and T15 were detected in swimming pool water samples in Alexandria [60]. Worldwide, other genotypes of *Acanthamoeba* have been isolated in environmental samples in many preceding studies: T9 and T4 in Turkey [63]; T3-T5 in biofilms and dust from a clinic in Brazil [64]; and T4 in tap water and swab samples of intensive care units in India [2]. Recently, T5 with 98% homology to *A*. *lenticulata* was isolated from a water sample collected in a hospital in Nicaragua [65]. T3-T5 and T11 were detected in tap water samples, and T4-5 and T16 in soil and tap water in Iran and China, respectively [66, 67], and genotypes T3-5, T7, T9-11, and T15-16 were isolated in Tunisia from oasis water used in various human activities [68]. The variance in the spreading of *Acanthamoeba* genotypes between studies could be attributed to the seasonal variation at the time of sampling which may encourage the occurrence of specific genotypes [69].

Notably, genotype T4 is considered the most common *Acanthamoeba* genotype in the environment worldwide and the leading causative agent of *Acanthamoeba* keratitis and GAE [21, 63, 70]. Moreover, compared to other *Acanthamoeba* genotypes, T4 showed significantly higher binding and yielded severe damage on host cells [71]. *A*. *astronyxis* (T7) was isolated from environmental sources by Booton *et al*. (2005) [72] and Dendana *et al*. (2018) [68]. However, there have been few studies that correlate this genotype with clinical cases. *A*. *astronyxis* was isolated from a corneal scrape of an AK case in Spain [73]. Furthermore, in Giza, Egypt, genotyping of two representative *Acanthamoeba* isolates from patients with keratitis confirmed their identity as *A*. *astronyxis* belonging to T7 [50].

Concerning the potential pathogenicity of the other morphologically identified *Acanthamoeba* species, as reported in the present study, different studies have emphasized their importance as human pathogens causing either keratitis or GAE, including *A*. *polyphaga* [74, 75], *A*. *culbertsoni* (T10) [76, 77], *A*. *triangularis* (T4) [39, 78], *A*.* lenticulata* (T5) [65, 70, 79–81], and *A*. *comandoni* (T9) [82]. For the first time, regarding Vahlkampfiidae, one isolate was identified as a member of the *Allovahlkampfia* genus using morphological and molecular methods. This is the only isolate that was successfully sequenced and genotyped. Homology and phylogenetic analyses confirmed that the strain belonged to this genus. However, owing to the shortage of available sequences for this genus in the GenBank database, classification at the species level was not definite. The isolate was established to belong to *Allovahlkampfia* species, showing 95.4% homological identity with *A*. *spelaea* isolated from the hemodialysis unit in Iran (accession number KY575234). It also showed 80.3% identity with the *A*. *spelaea* strain SK1 (accession number EU696949). Interestingly, a closely related species was identified for the first time as a causal agent for chronic keratitis in an Egyptian patient by Tolba *et al*. (2016) [9]. Furthermore, the *Allovahlkampfia* genus was first isolated from dishcloths and in the Spanish territory by Reyes-Batlle *et al*. (2019) [62]. Regarding the possible pathogenicity of *Vahlkampfia*, different studies have confirmed their ability to be pathogenic or even to act as carriers of other pathogenic agents [83]. Two cases of encephalitis, likely due to *Vahlkampfia*, have been reported based only on morphology [84]. *Vahlkampfia* keratitis was also reported in contact lens and non-contact lens wearers following minor corneal trauma [85]. Other keratitis cases due to mixed infection of *Acanthamoeba* and *Vahlkampfia* in Iran and Spain were recorded [86, 87].

Therefore, our results highlight the clinico-epidemiological importance of FLA, which can act as pathogens and vehicles of other pathogenic agents. The presence of FLA in water used for different domestic activities could represent a risk source for FLA and amoeba-resistant pathogens. Consequently, appropriate processing of water is essential.

## Conclusion

To the best of our knowledge, this is the first report presenting the occurrence of potentially pathogenic *Acanthamoeba* and Vahlkampfiidae in treated potable domestic water in Assiut City, Upper Egypt. Genotyping of *Acanthamoeba* revealed the presence of T4 and T7 genotypes that represent a risk for human health. In addition, the newly detected Vahlkampfiidae, including *Vahlkampfia* and *Allovahlkampfia*, need additional workup and phylogenetic analysis to better understand the genotypic diversity of these amoebae and their pathogenicity using larger sample sizes within different environmental sources. Our findings should raise awareness among clinicians and public health specialists to consider the related health hazards of these amoebae for human beings.

## Supporting information

S1 Fig Agarose gel 1.5% stained with ethidium bromide showing the negative PCR reaction of 4 samples of *Naegleria*, Lanes (3, 5–7), failed to amplify ITS region using species-specific primers of *Naegleria fowleri*, lanes 2 and 4 = negative controls, and lane 1 (M) marker = 100 bp DNA ladder. (TIF)

S2 Fig Distance matrix of *Acanthamoeba* sp. sequences. (TIF)

S3 Fig Distance matrix of *Allovahlkampfia* sp. sequences. (TIF)

S1 Table Sampling sites in Assiut City. (DOCX)

S2 Table Isolates used for the construction of a phylogenetic tree and distance matrix. (DOCX)

S3 Table Seasonal variation of the prevalence of FLA, *Acanthamoeba*, and Vahlkampfiidae in different water samples. (DOCX)

S1 Raw images (PDF)

S1 File Filter apparatus designed by Faculty of Engineering workshops. (DOCX)

S2 File *Acanthamoeba* sp. sequences matrix. (TXT)
