# Supplementary figures and images for: Isolation and morphological and molecular characterization of waterborne free-living amoebae: Evidence of potentially pathogenic Acanthamoeba and Vahlkampfiidae in Assiut, Upper Egypt
Source: PLoS One. 2022 Jul 8;17(7):e0267591. doi: 10.1371/journal.pone.0267591 (PMC9269480; doi:10.1371/journal.pone.0267591)

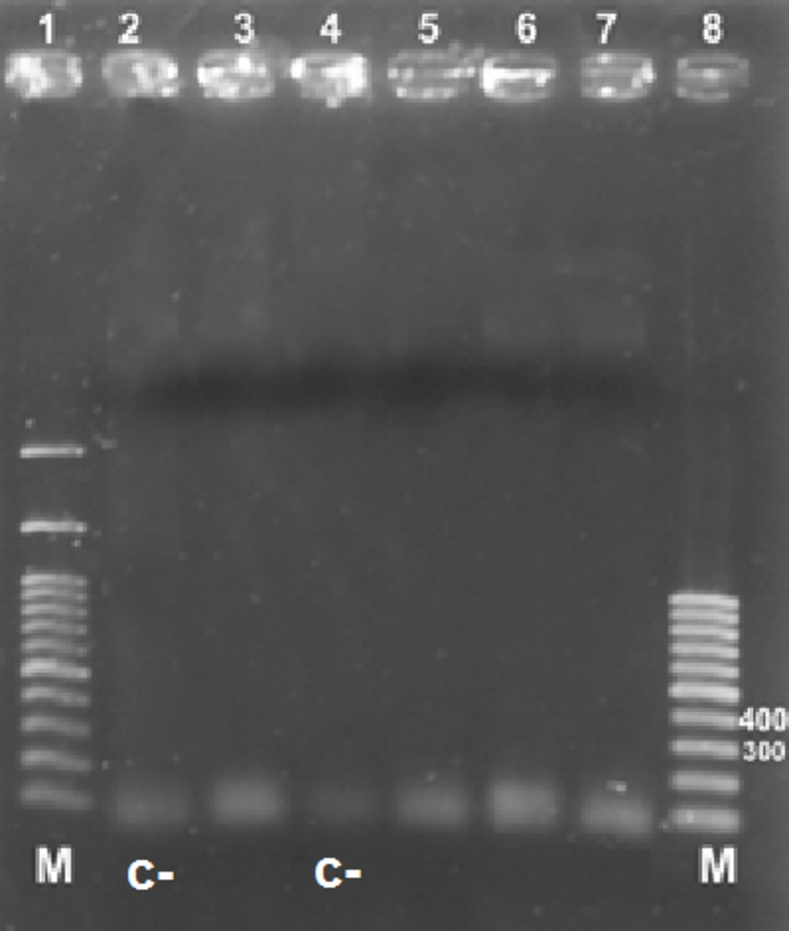

Supplement: S1 Fig — (TIF) [file pone.0267591.s001.tif]

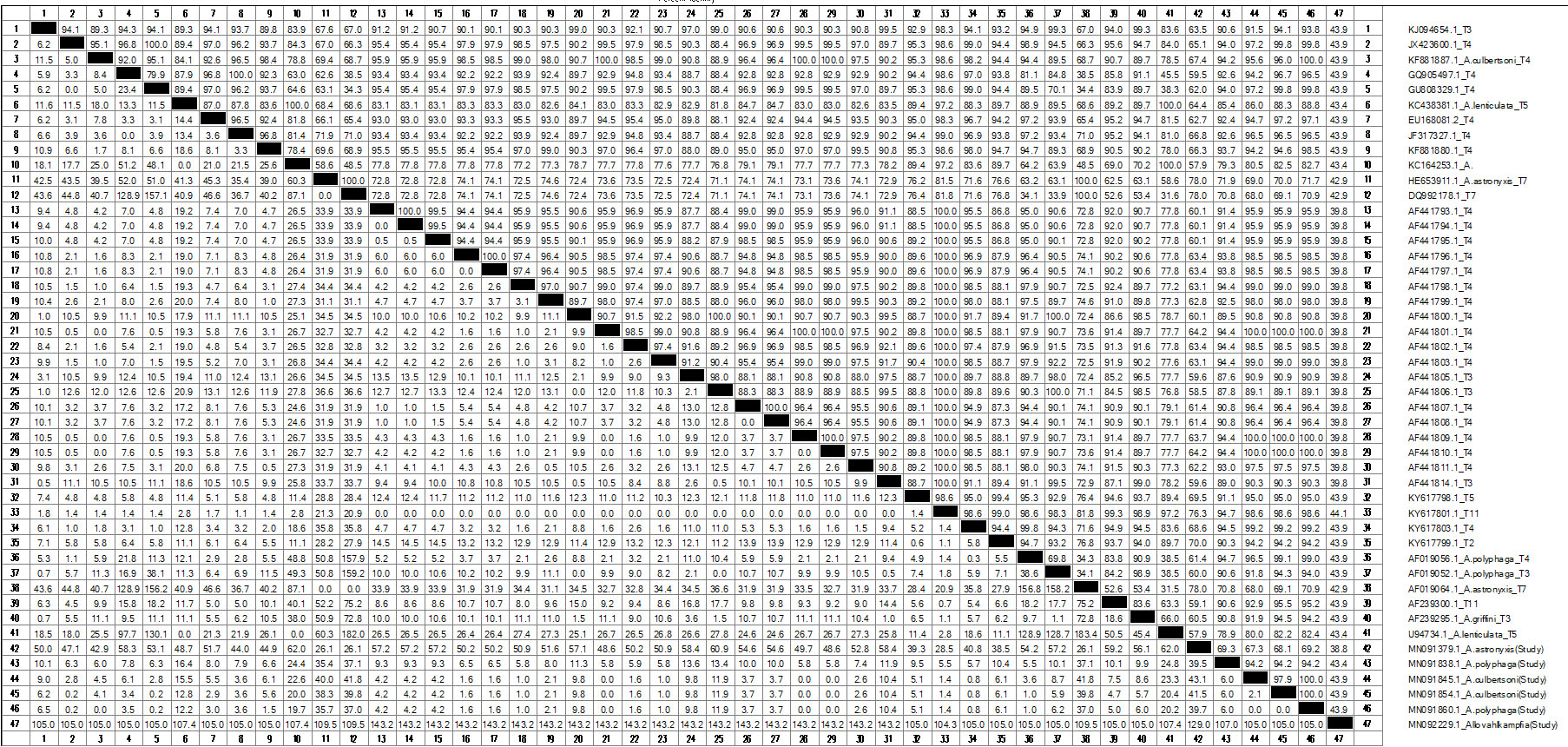

Supplement: S2 Fig — (TIF) [file pone.0267591.s002.tif]

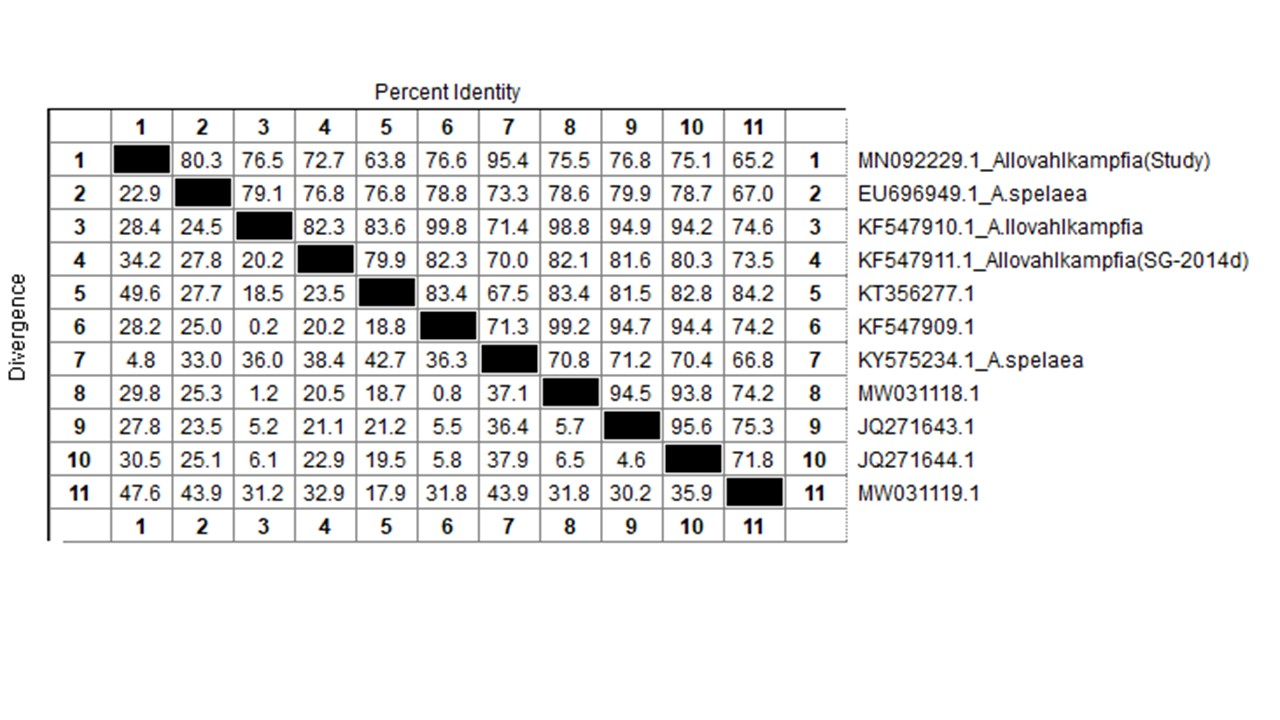

Supplement: S3 Fig — (TIF) [file pone.0267591.s003.tif]
