# Supplementary material for: Isolation and morphological and molecular characterization of waterborne free-living amoebae: Evidence of potentially pathogenic Acanthamoeba and Vahlkampfiidae in Assiut, Upper Egypt
Source: PLoS One. 2022 Jul 8;17(7):e0267591. doi: 10.1371/journal.pone.0267591 (PMC9269480; doi:10.1371/journal.pone.0267591)
Supplement: S1 Table — (DOCX) [file pone.0267591.s004.docx]

Tap water sampling sites in Assiut City.

| **Region** | **Samples No.** |
| --- | --- |
| **Sherkt Fryal**  Mahmoud Rashwan street  Imam Ali street | 4*  4 |
| **Sherkt Qolta**  King Seti street  Mohamed Ali Makarm street | 4  4 |
| **Al-Arbaeen**  Mobarak City  El-Lewa Abd El-Haleem Mousa street | 4  4 |
| **Al-Hamra**  Gisr Al-Saliba street  Al-Fadaa street | 4  4 |
| **Al-Walidya**  Taksim Al-Rai street  El-Nasr street | 4  4 |
| **El-Sadat**  Gisr Al-Soltan street  Taksim Al-Gindi street | 4  4 |
| **Al-Moalmeen**  Mohammed Abd El-Aziz street  Al-Oteify street | 4  4 |
| **Seed region**  Besery way 1  Besery way 2 | 4  4 |
| **Taksim Al-Hqoqeen**  Blal Bn Rbah street  Taksim Al-Hqoqen street no.1 | 4  4 |
| Al-Majzoub square | 4 |
| Al-Shader | 4 |
| Alot Al-Nasara street | 4 |
| Ezzat Galal street | 4 |
| Al-Nemeis street | 4 |
| El-Gomhoryaa street | 4 |
| El-Helaly street | 4 |
| Total | 100 |

*One sample each season

Tank water sampling sites in Assiut City.

| **Region** | **Samples No.** |
| --- | --- |
| Al-Walidya1 | 4* |
| Al-Walidya2 | 4 |
| El-Sadat | 4 |
| Gamal Abd Elnaser street | 4 |
| El-Maamoun street | 4 |
| King Seti street 1 | 4 |
| King Seti street 2 | 4 |
| Ryad street | 4 |
| Al-Nemeis | 4 |
| Al-Majzoub square | 4 |
| El-Gomhoryaa street 1 | 4 |
| El-Gomhoryaa street 2 | 4 |
| El-Helaly street | 4 |
| Taksim El-Hoqoqeen 1 | 4 |
| Taksim El-Hoqoqeen 2 | 4 |
| Yousry Ragib street | 4 |
| Palestine street | 4 |
| Al-Moalmeen 1 | 4 |
| Al-Moalmeen 2 | 4 |
| 23 Youlio street | 4 |
| Total | 80 |

Swimming pools included in the present study.

| **Swimming pools** | **Samples No.** |
| --- | --- |
| Assiut University Olympic Village pool | 4* |
| Assiut University Faculty of Physical Education pool | 4 |
| Total | 8 |
