# Supplementary material for: Isolation and morphological and molecular characterization of waterborne free-living amoebae: Evidence of potentially pathogenic Acanthamoeba and Vahlkampfiidae in Assiut, Upper Egypt
Source: PLoS One. 2022 Jul 8;17(7):e0267591. doi: 10.1371/journal.pone.0267591 (PMC9269480; doi:10.1371/journal.pone.0267591)
Supplement: S2 Table — (DOCX) [file pone.0267591.s005.docx]

| Isolates used for the construction of a phylogenetic tree and distance matrix. | | | | |
| --- | --- | --- | --- | --- |
| **Accession** | **Name** | **source** | **Genotype** | **Country** |
| KJ094654 | Acanthamoeba isolate AcaP 18 | Water | T3 | Cairo, Egypt |
| JX423600 | Acanthamoeba genotype T4 voucher  OSU08-014 | Water | T4 | Cairo, Egypt |
| KF881887 | Acanthamoeba culbertsoni strain It-N | Water | T4 | Cairo, Egypt |
| GQ905497 | Acanthamoeba spp. AcaVNAK03 | Water | T4 | Cairo, Egypt |
| GU808329 | Acanthamoeba spp. Ac_PCN20 | Water |  | Cairo, Egypt |
| KC438381 | Acanthamoeba lenticulata isolate  33195463 | Soil | T5 | Cairo, Egypt |
| EU168081 | Acanthamoeba spp. UIC 1060  voucher OSU 06-033 | Soil | T4 | Cairo, Egypt |
| JF317327 | Acanthamoeba spp. MN/TRW14/IRN | Soil | T4 | Cairo, Egypt |
| KF881880 | Acanthamoeba spp. strain md-H | Soil | T4 | Cairo, Egypt |
| KC164253 | Acanthamoeba lenticulata clone  CF1-249 | Soil | T5 | Cairo, Egypt |
| HE653911 | Acanthamoeba astronyxis S1 | Keratitis | T7 | Cairo, Egypt |
| DQ992178 | Acanthamoeba astronyxis EFW | Keratitis | T7 | Cairo, Egypt |
| AF441793.1 | Acanthamoeba sp. isolate P97RCLS1 | Cornea/lens | T4 | Hong Kong |
| AF441794.1 | Acanthamoeba sp. isolate P97LCSS1 | Cornea/lens | T4 | Hong Kong |
| AF441795.1 | Acanthamoeba sp. isolate P97LCLS2 | Cornea/lens | T4 | Hong Kong |
| AF441796.1 | Acanthamoeba sp. isolate P97RCLS2 | Cornea/lens | T4 | Hong Kong |
| AF441797.1 | Acanthamoeba sp. isolate P97LCLS1 | Cornea/lens | T4 | Hong Kong |
| AF441798.1 | Acanthamoeba sp. isolate C124LC | Lens | T4 | Hong Kong |
| AF441799.1 | Acanthamoeba sp. isolate C10TA | Faucet | T4 | Hong Kong |
| AF441800.1 | Acanthamoeba sp. isolate P77LC | Lens | T3 | Hong Kong |
| AF441801.1 | Acanthamoeba sp. isolate P191CL | Lens | T4 | Hong Kong |
| AF441802.1 | Acanthamoeba sp. isolate P209CS | Corneal scraping | T4 | Hong Kong |
| AF441803.1 | Acanthamoeba sp. isolate C68TA | Faucet | T4 | Hong Kong |
| AF441805.1 | Acanthamoeba sp. isolate P208TA | Faucet | T3 | Hong Kong |
| AF441806.1 | Acanthamoeba sp. isolate P191TA | Faucet | T3 | Hong Kong |
| AF441807.1 | Acanthamoeba sp. isolate P91LC | Lens | T4 | Hong Kong |
| AF441808.1 | Acanthamoeba sp. isolate P91CS | Corneal scraping | T4 | Hong Kong |
| AF441809.1 | Acanthamoeba sp. isolate P120CL | Lens | T4 | Hong Kong |
| AF441810.1 | Acanthamoeba sp. isolate P120CS | Corneal scraping | T4 | Hong Kong |
| AF441811.1 | Acanthamoeba sp. isolate P6CS | Corneal scraping | T4 | Hong Kong |
| AF441814.1 | Acanthamoeba sp. isolate P120TA | Faucet | T3 | Hong Kong |
| KY617798.1 | Acanthamoeba genotype T5 isolate aabb7 | Water | T5 | Iran |
| KY617801.1 | Acanthamoeba genotype T11 isolate aabb10 | Water | `T11 | Iran |
| KY617803.1 | Acanthamoeba genotype T4 isolate aabb12 | Water | T4 | Iran |
| KY617799.1 | Acanthamoeba genotype T2 isolate aabb8 | Water | T2 | Iran |
| KY587116.1 | Acanthamoeba genotype T4 isolate aabb4 | Water | T4 | Iran |
| AF019056.1 | Acanthamoeba polyphaga HC-2 | Water | T4 | Mexico |
| AF019052.1 | Acanthamoeba polyphaga Panola Mountain | Soil | T3 | USA |
| AF019064.1 | Acanthamoeba astronyxis Ray & Hayes | Water | T7 | USA |
| AF239300.1 | Acanthamoeba sp. Dargon strain | Keratitis | T11 | UK |
| AF239295.1 | Acanthamoeba griffini strain CCAP | Water | T3 | USA |
| U94734.1 | Acanthamoeba lenticulata strain 407-3a | Water | T5 | USA |
