# Supplementary material for: Isolation and morphological and molecular characterization of waterborne free-living amoebae: Evidence of potentially pathogenic Acanthamoeba and Vahlkampfiidae in Assiut, Upper Egypt
Source: PLoS One. 2022 Jul 8;17(7):e0267591. doi: 10.1371/journal.pone.0267591 (PMC9269480; doi:10.1371/journal.pone.0267591)
Supplement: S3 Table — (DOCX) [file pone.0267591.s006.docx]

Seasonal variation of the prevalence of FLA, *Acanthamoeba*, and Vahlkampfiidae *(Naegleria like)* in different water samples

|  | Results | Season  N=188 | | | | | | | | *P*-value |
| --- | --- | --- | --- | --- | --- | --- | --- | --- | --- | --- |
|  |  | **Autumn**  **N=47** | | **Winter**  **N=47** | | **Spring**  **N=47** | | **Summer**  **N=47** | |  |
|  |  | **No.** | **%** | **No.** | **%** | **No.** | **%** | **No.** | **%** |  |
| FLAs | **Positive** | 13 | 27.6 | 7 | 18.4 | 14 | 29.8 | 18 | 38.2 | 0.086 |
|  | **Negative** | 34 | 72.4 | 40 | 81.6 | 33 | 70.2 | 29 | 61.8 |  |
| *Acanthamoeba* | **Positive** | 11* | 27.6 | 6* | 18.4 | 11* | 27.6 | 11* | 34.1 | 0.193 |
|  |  | 2** |  | 1** |  | 2** |  | 5** |  |  |
|  | **Negative** | 34 | 72.4 | 40 | 81.6 | 34 | 72.4 | 31 | 65.9 |  |
| Vahlkampfiidae  (*Naegleria* like) | **Positive** | 2** | 4.3 | 1** | 2.1 | 1* | 6.4 | 2* | 14.9 | 0.076 |
|  |  |  |  |  |  | 2** |  | 5** |  |  |
|  | **Negative** | 45 | 95.7 | 46 | 97.9 | 44 | 93.6 | 40 | 85.1 |  |

* Single strain in the water sample (either *Acanthamoeba* or Vahlkampfiidae)

** Mixed isolates of both *Acanthamoeba* and Vahlkampfiidae *(Naegleria* like) in the same water sample
