# Supplementary material for: Isolation and morphological and molecular characterization of waterborne free-living amoebae: Evidence of potentially pathogenic Acanthamoeba and Vahlkampfiidae in Assiut, Upper Egypt
Source: PLoS One. 2022 Jul 8;17(7):e0267591. doi: 10.1371/journal.pone.0267591 (PMC9269480; doi:10.1371/journal.pone.0267591)

S1 Raw images:

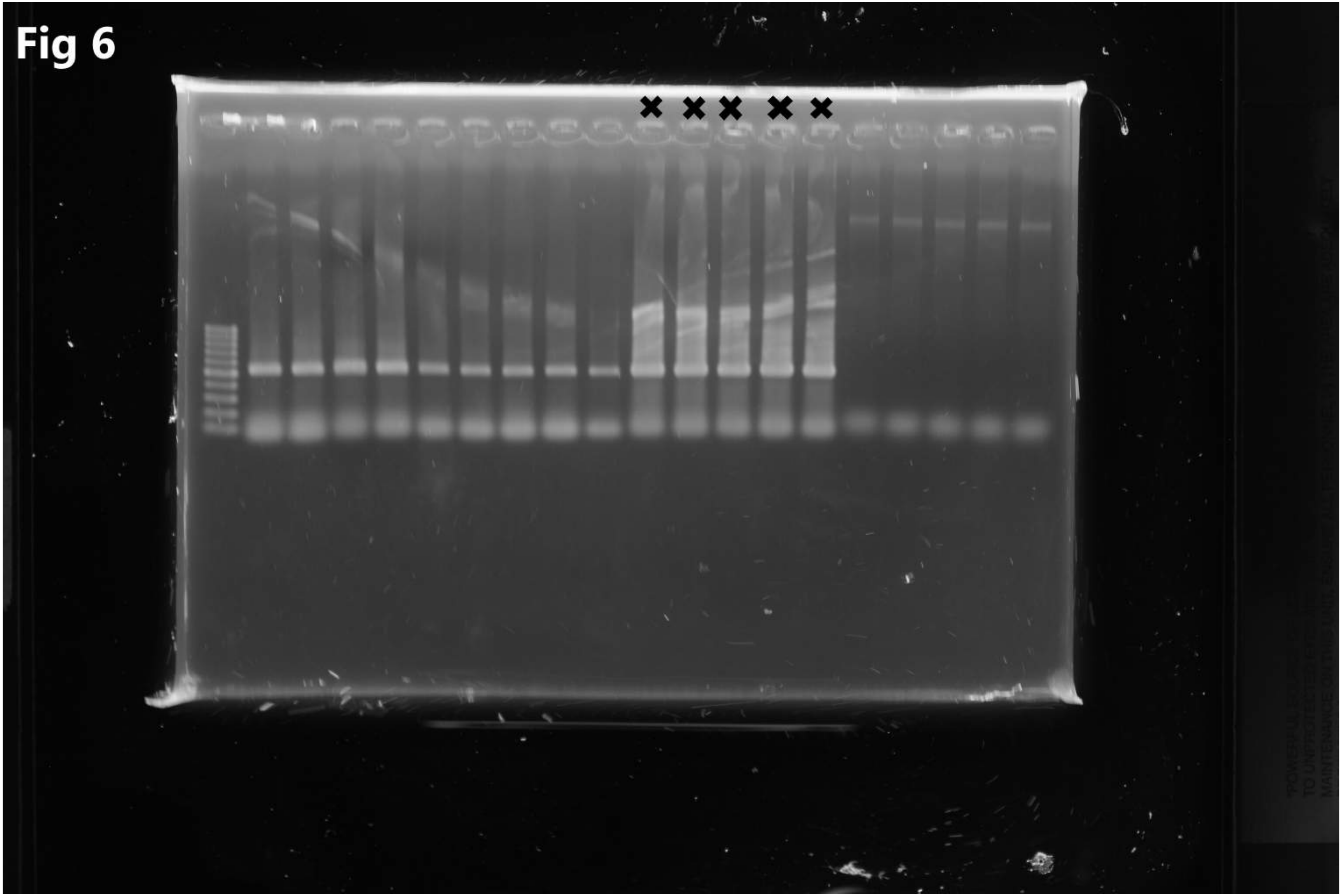

Uncropped image of Fig 6: The only modification is cropping the image to exclude marked lanes with x which were repeated samples.

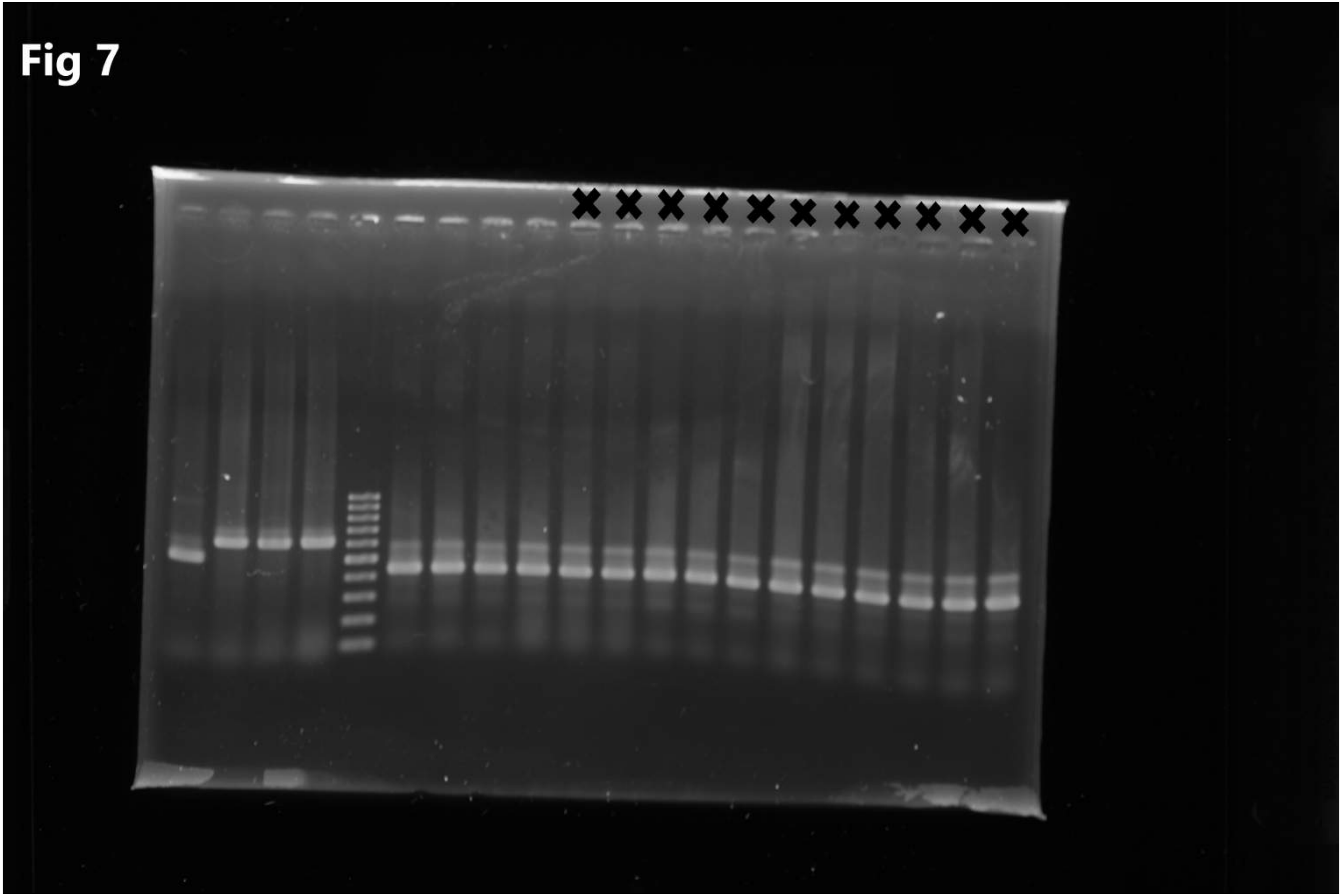

Uncropped image of Fig 7: The only modification is cropping the image to exclude marked lanes with x which were repeated samples.

S1 Fig

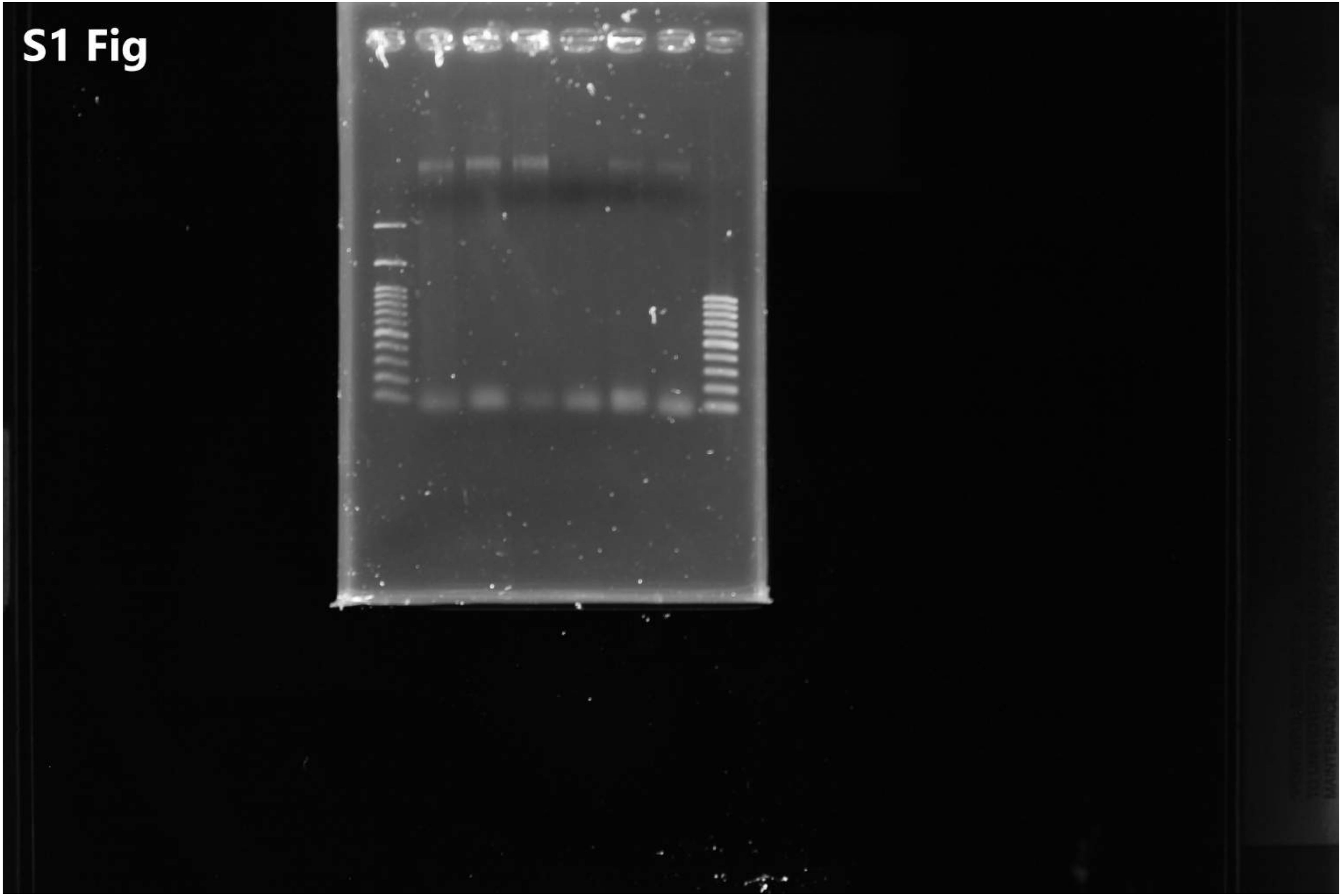

Supplement: S1 Raw images — (PDF) [file pone.0267591.s007.pdf]
