# Supplementary material for: Isolation and morphological and molecular characterization of waterborne free-living amoebae: Evidence of potentially pathogenic Acanthamoeba and Vahlkampfiidae in Assiut, Upper Egypt
Source: PLoS One. 2022 Jul 8;17(7):e0267591. doi: 10.1371/journal.pone.0267591 (PMC9269480; doi:10.1371/journal.pone.0267591)
Supplement: S1 File — (DOCX) [file pone.0267591.s008.docx]

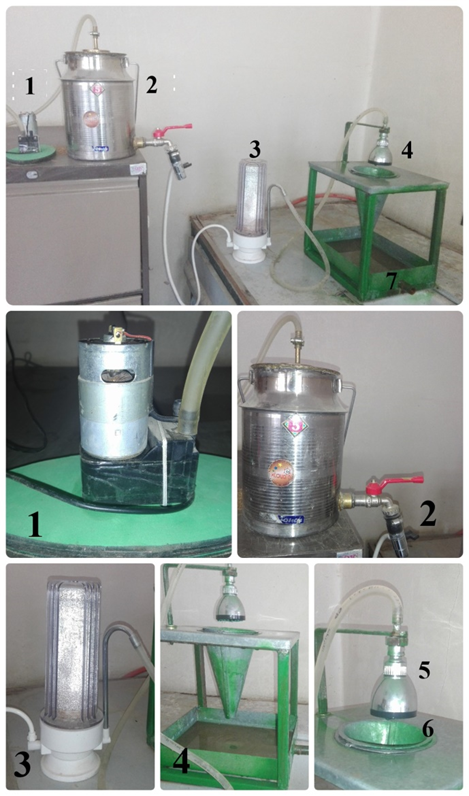


Parts of water filter apparatus designed by Faculty of Engineering workshops: ^(1)^ Vacuum Air Pump; ^(2)^ stainless steel tank; ^(3)^ filter cartridge; ^(4)^ filtration drainage box; ^(5)^ shower faucet ^(6)^ membrane filter housing and ^(7)^ drainage sink.

**The apparatus consists of:**

1. Vacuum air pump
2. Three liters stainless steel tank placed at 50cm height
3. The tank is connected to a filter cartridge with a pore size of 100µm to remove large particles and debris.
4. The filter cartridge is connected to filtration-drainage box containing a shower faucet to distribute water equally over the membrane filter.
5. A stroge housing, closely fitted for nitrocellulose membrane filter (0.45 µm pore size and 47 mm diameter) (Whatman, WCN type, Cat No. 7141-104).
6. The water passing through the membrane filter is drained into a nearby sink.

- The tank was cleaned with distilled water after each sample filtration process is completed. Distilled water was also passed through the filter cartridge to remove any FLA cysts that may be attached from the previous sample filtration process.
